# Exploring the potential factors of burning mouth syndrome in elderly people: A cross-sectional study

**DOI:** 10.4317/jced.64113

**Published:** 2026-05-29

**Authors:** André Barreiros, Ivan Albuquerque, Fabricio Almeida, Juliana Ramacciato, Luciana Oliveira

**Affiliations:** 1 São Leopoldo Mandic Institute and Research Center, Campinas, BrazilBurning Mouth Syndrome (BMS) remains a clinical challenge because of the unclear and multifactorial nature of its etiopathogenesis. This cross-sectional study aimed to determine the prevalence and potential factors involved in the occurrence of BMS in older adults.; 2 School of Dentistry, FINAMA, Belém, Brazil

## Abstract

**Background:**

Burning Mouth Syndrome (BMS) remains a clinical challenge because of the unclear and multifactorial nature of its etiopathogenesis. This cross-sectional study aimed to determine the prevalence and potential factors involved in the occurrence of BMS in older adults.

**Materials and Methods:**

Data from 365 individuals attending healthcare services in Manaus, Brazil, were analyzed. Clinical evaluation was conducted by a single calibrated examiner. Sociodemographic aspects, health conditions, medication use, smoking habits, need for dental prostheses, presence of xerostomia, and reports of BMS were also evaluated. The chi-square test, Fisher's exact test, G-test, binary logistic regression, and odds ratios were used to estimate the occurrence of BMS based on the independent variables.

**Results:**

The prevalence rate of BMS was 4.9%. In univariate analyses, BMS was significantly associated with race (p &lt; 0.001), diabetes mellitus (p = 0.013), systemic arterial hypertension (p = 0.024), and xerostomia (p = 0.005). In the binary logistic regression model, BMS was significantly associated with race (p = 0.031), with white participants having a 7.3 times higher risk of BMS than multiracial participants (OR = 7.3; 95% CI: 2.4-22.1). Binary logistic regression also showed that xerostomia was significantly associated with BMS (p = 0.019), increasing the risk by 3.8 times (OR = 3.8; 95% CI: 1.2-11.8).

**Conclusions:**

Race and xerostomia were associated with BMS in older adults. These findings may help clinicians refine and tailor treatment strategies within a personalized care approach for this population.

## Introduction

Burning mouth syndrome (BMS) is defined by the World Health Organization as a chronic orofacial pain characterized by an intraoral or dysesthetic burning sensation that recurs for more than 2 h a day on at least 50% of days for more than 3 months, with no evident causative lesions found upon investigation and clinical examination (1). The term "syndrome" is used when multiple subjective symptoms are reported simultaneously, rather than an isolated single isolated complaint. Commonly reported symptoms include xerostomia, hyposalivation, dysgeusia, a paper-like sensation, or a swelling sensation (2). The condition is more common in patients with anxiety and depression who tend to somatize, affecting various structures of the stomatognathic system, including the tongue, lips, oral mucosa, and tongue mucosa (3). A previous systematic review evaluated the relationship between thyroid disorders and BMS. The authors concluded that thyroid hormone abnormalities are a factor in secondary BMS, especially in patients with hypothyroidism (4). Another systematic review assessed the worldwide prevalence and epidemiological profile of BMS, finding an overall pooled prevalence of 1.73% in the general population and 7.72% in clinical patients. Moreover, subgroup analysis by age showed that prevalence was higher in people over 50 (3.31%) than in those under 50 (1.92%) (5). Age-related diseases have become more prevalent as societies have aged, and pathological changes in the elderly challenge health care systems. A previous study reported that older adults are generally considered frequent users of medicines due to multiple chronic diseases. The authors concluded that medications may play an important role in the development of BMS (6). Based on previous information, it remains unclear which etiopathogenic factors are responsible for BMS, and treating this condition continues to be a clinical challenge (2 , 7). Recent evidence-based guidelines recommend an individualized approach to care, taking patient-specific factors into account (8). Moreover, epidemiological evidence is limited, with most focusing on elderly populations (9 - 11). A global systematic review and meta-analysis further highlighted that racial and ethnic disparities in BMS have not been widely studied (5). A previous systematic review found that BMS patients reported lower overall quality of life and oral health-related quality of life than control groups, highlighting the impact of this condition on patients' well-being (12). In this context, estimating the prevalence of BMS in older adults is essential to generate further evidence on associated factors and inform the planning of healthcare strategies and priorities for this population.

## Materials and Methods

- Study design and ethical approval This cross-sectional study was conducted in accordance with established guidelines for reporting observational studies (13). The study was approved by the Ethics Committee of the São Leopoldo Mandic Institute and Research Center under registration number 5.237.120, Campinas, São Paulo, Brazil. All participants provided written informed consent before their data were used. - Participants and data collection The criteria for establishing the sample's representativeness were based on scientific articles on the same topic, which determined the number of interviews and examinations to be conducted among the research participants. The inclusion criteria were as follows: age 60 years or older and attendance at elderly healthcare support units located in the municipality of Manaus, in northern Brazil. The exclusion criteria were symptoms of dental or systemic origin, lesions in the mouth, and unwillingness to sign the informed consent term. The sample consisted of 365 elderly individuals selected through non-probabilistic, intentional, and accidental sampling, with a median age of 68 years. Participation was voluntary, and all individuals received a clear explanation of the study objectives and the informed consent form, with assurance of confidentiality and anonymity. The research protocol for the selected participants consisted of an intraoral clinical evaluation conducted by a single calibrated examiner using both artificial and natural light. The process included completing a specific medical history and physical examination form for research purposes, as well as an interview. The data collected included sociodemographic information, overall health conditions, medication use, smoking status, need for dental prostheses, presence of xerostomia, and reports of BMS. BMS was assessed using the Douleur Neuropathique en 4 Questions (DN4) questionnaire (14 , 15). The instrument was translated into Portuguese by the Faculty of Medicine of the University of Porto in 2007, with authorization from its author, Didier Bouhassira. The DN4 was administered in conjunction with the Visual Analog Scale (VAS) for pain (16 , 17), a 0-10 scale in which 0 indicates no pain and 10 represents the worst imaginable pain. The VAS was applied to participants presenting symptoms of BMS. - Statistical analyses Descriptive analyses were initially performed for both the overall sample and by subgroup. Sociodemographic aspects, health conditions, medication use, smoking habits, need for dental prostheses, presence of xerostomia, and BMS are presented as absolute frequencies (n) and relative percentages (%). Associations were evaluated using the chi-square test and Fisher's exact test. All analyses were conducted using R software (R Core Team, 2020), with a significance level set at 5%. Univariate analyses were performed using the Chi-square test, Fisher's exact test, and G-test to investigate associations between sociodemographic factors, health conditions, medication use, smoking habits, dental prosthesis use, and xerostomia with the dependent variable BMS. Binary logistic regression and odds ratios (OR) were used to estimate the occurrence of BMS based on independent variables, which were included in the model if they had a p-value &lt; 0.20. Variables that remained in the final model were identified using the Wald method (p &lt; 0.05). Statistical analyses were conducted using SPSS 23 (SPSS Inc., Chicago, IL, USA), with a significance level of 5%.

## Results

This study included 365 participants: 111 (30.4%) male and 254 (69.6%) female. Participants' ages ranged from 60 to 91 years, with a mean age of 68.4 years, a standard deviation of 5.1 years, and a median age of 68 years. Most participants were multiracial (n = 222; 60.8%) or white (n = 74; 20.3%), while other racial categories and blank responses accounted for 13.3% (n = 49) and 5.5% (n = 20), respectively, as shown in Table 1. Of the 365 participants, 72 (19.7%) had diabetes mellitus, 132 (36.2%) had systemic arterial hypertension, and 52 (14.2%) had heart disease. Other health conditions were reported by 195 (53.4%) participants, and 314 (86.0%) participants indicated routine medication use (Table 1).

Table 1

**Figure 1:**
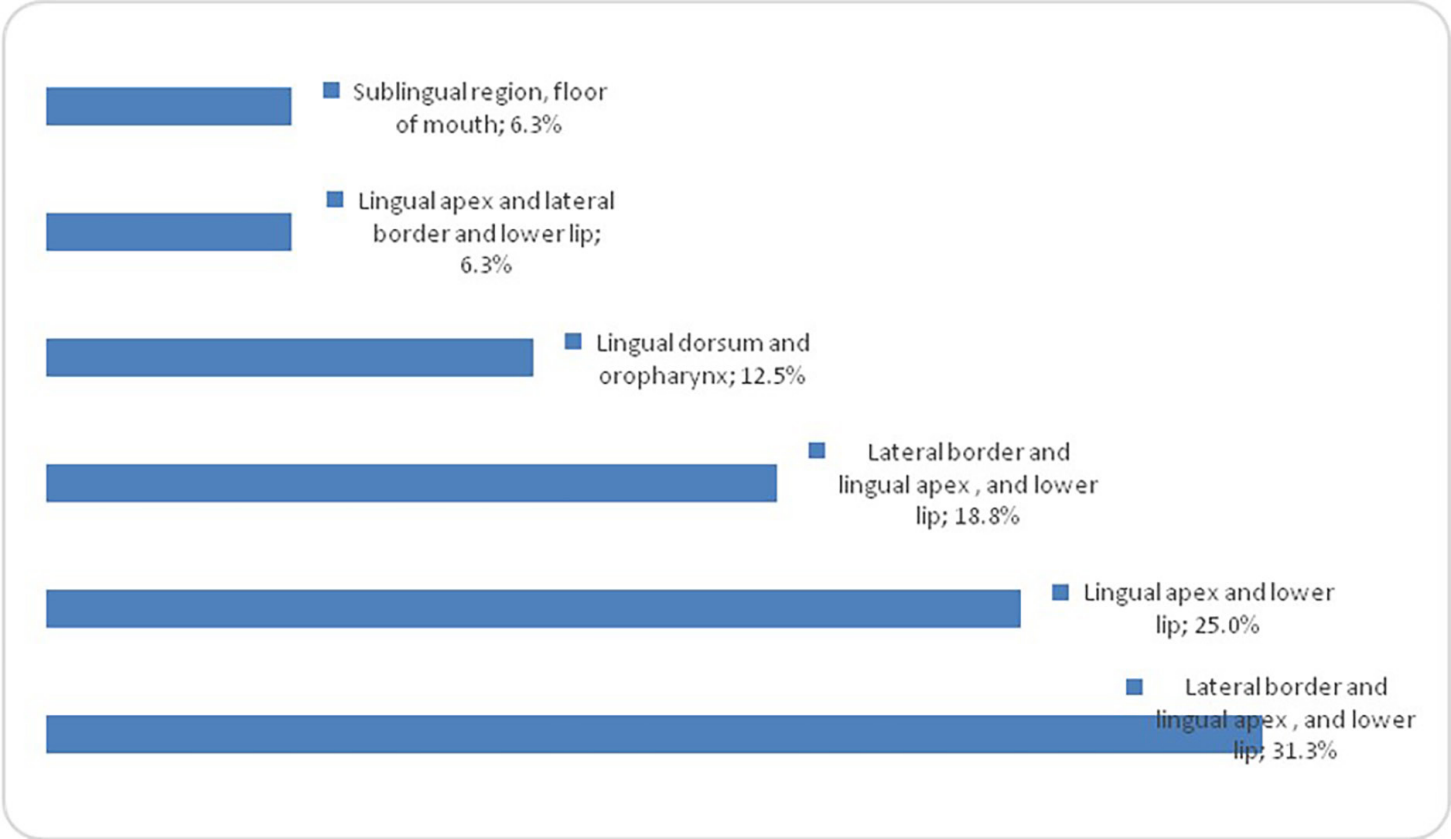
Distribution of mouth sites where participants reported feeling burning.

Current smoking was reported by 13 (3.6%) participants, and 104 (28.5%) identified themselves as former smokers, as shown in Table 1. In the sample, 176 (48.2%) participants had no dental prostheses. Among users, 93 (25.5%) had complete prostheses and 107 (29.3%) had partial prostheses. Concomitant use of complete and partial dental prostheses was reported by 41 (11.2%) participants. Xerostomia was reported by 176 (48.2%) participants. The prevalence rate of BMS was 4.9%, with duration ranging from 1.5 to 5 years and an average of 2.8 years, with a standard deviation of 1.0 year (Table 1). All BMS cases in the sample, except for one with no response, were secondary, with intermittent duration in 93.3% of the 15 cases with responses. Figure 1 shows the sites where participants who answered the questionnaire reported experiencing burning.

1

**Figure 2:**
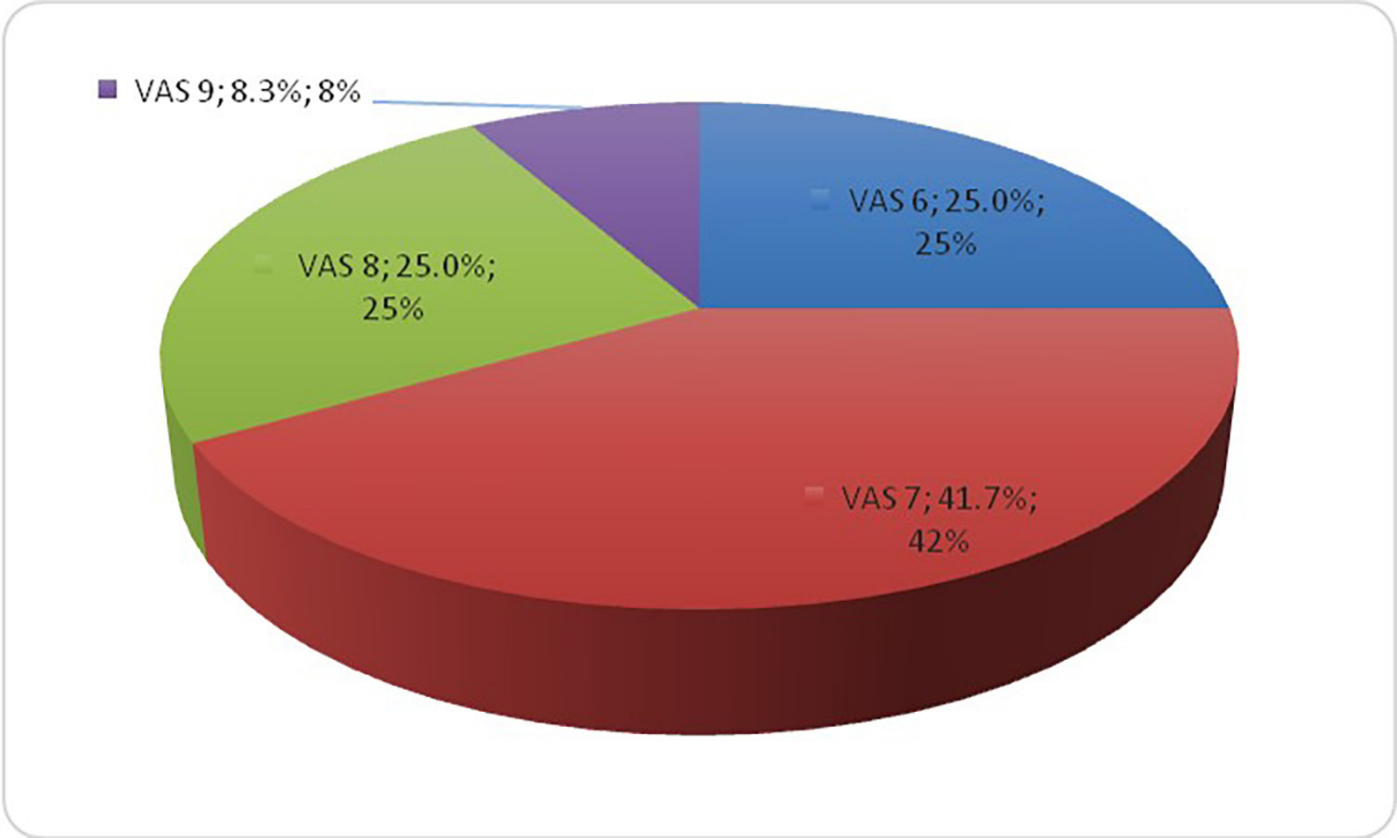
VAS scale of symptoms of burning mouth syndrome ranging from 0 to 10.

The burning sensation was felt exclusively in the morning, afternoon, or evening by 3 (18.7%) of the 16 participants. One (6.3%) reported burning in both the morning and afternoon. Three (18.7%) participants experienced burning in the morning and evening, and 8 (50.0%) felt burning in the afternoon and evening. One (6.3%) participant indicated that it occurred in the morning, afternoon, and evening. The univariate analyses indicated that BMS was significantly associated with race (p &lt; 0.001), diabetes mellitus (p = 0.013), systemic arterial hypertension (p = 0.024), and xerostomia (p = 0.005) (Table 1). Surprisingly, the binary logistic regression model (Table 1) showed that BMS was significantly associated with race (p = 0.031), with white participants having a 7.3 times higher risk of BMS than multiracial participants (OR = 7.3; 95% CI: 2.4-22.1). Binary logistic regression also showed that xerostomia was significantly associated with BMS (p = 0.019), increasing the risk by 3.8 times (OR = 3.8; 95% CI: 1.2-11.8). Of the 18 BMS patients, 15 received the DN4 classification, with Neuro03 for 1 (6.7%) participant and Neuro04 for the other 14 (93.3%). Pain was reported by 12 of the 18 cases, with VAS scores ranging from 6 to 9; most participants indicated a pain level of 7 (Fig. 2).

2

In addition, food was the most frequently indicated triggering factor, while water, ice, and cold food were the most commonly mentioned recovery factors.

## Discussion

This study provides a comprehensive analysis of BMS in older adults by examining not only its prevalence but also its potential associated factors, including age, gender, and certain racial and ethnic characteristics. A prevalence rate of 4.9% was observed based on the BMS diagnostic criteria. A prior study found an overall pooled worldwide prevalence of BMS of 1.73% in the general population (5). In a previous observational study, the stomatological conditions of more than 5,000 elderly patients were reviewed, with BMS being the most prevalent condition (10). Our results showed that the most frequently affected sites were the tongue and lower lips. In the study by Steele et al. (18), the most frequently affected sites were the tongue, lips, palate, throat, and oral mucosa. Accurate diagnosis of BMS in older adults is essential, particularly as the prevalence of age-related conditions continues to rise and place increasing demands on healthcare systems. Age-related changes in the oral cavity reflect not only physiological alterations but also factors such as tooth loss without prosthetic rehabilitation, as well as the effects of systemic diseases, comorbidities, and medication use. Clinicians should be aware of the potential consequences of BMS and be prepared to manage affected patients appropriately. The present findings may also help inform the development of targeted health promotion strategies for this population. Previous studies have shown that patients with BMS report poor health-related quality of life and oral health-related quality of life (12 , 19). In addition, BMS has been associated with psychiatric and anxiety disorders (20 , 21). Comorbidities are also common; Steele et al. (18) reported conditions such as controlled hypertension, diabetes mellitus, history of hypothyroidism, depression, anxiety, and treated chronic diseases. In this study, 8 individuals with BMS (44.4%) had diabetes mellitus, 11 (61.1%) had systemic arterial hypertension, and 5 (27.7%) had heart disease. Other health conditions were reported by 12 (66.6%) participants, consistent with the results presented by the mentioned authors, with BMS patients being 3.58 times more likely to develop gastrointestinal problems than people without BMS, especially gastritis, esophageal reflux, and flatulence. The use of multiple medications, antihypertensives, and benzodiazepines was associated with BMS (6 , 21 - 23). In this study, all participants with BMS used some type of routine medication. Antihypertensives were used by ten participants and antihyperglycemics by six participants at the time of the exam. In the present sample, disease duration ranged from 1.5 to 5 years, with an average of 2.8 years and a standard deviation of 1.0 year, similar to the 2.7-year average reported by Steele et al. (18). According to the DN4 questionnaire, 18 participants were classified as Neuro03 or Neuro04. Pain intensity was reported by 12 of the 18 BMS cases, with VAS scores ranging from 6 to 9 on a 0-10 scale; most participants rated their pain as 7. The mean VAS score in this study was 6.64 (± 1.85), compared with 5.9 (± 1.9) reported by Brailo et al. (15). Most participants with BMS were women. Previous studies have shown that BMS is more common in middle-aged and elderly women, with a ratio of 3:1 (24). The literature indicates that BMS occurs in an approximate age range of 60 to 80 years, with an average age of 60 years (9 , 15). In this study, participants were aged between 60 and 91 years, with an average of 68.4 years, a standard deviation of 5.1 years, and a median of 68 years, data that corroborate previous studies (15 , 25). Women affected by BMS are usually menopausal or postmenopausal, with an average age of 55 to 60 years (3 , 5 , 24). A systematic review concluded that females have a higher risk of developing BMS than males due to sleep disorders, personality traits, and biopsychosocial changes (26). However, a recent study recommended further investigation of hormonal alterations, which may be a promising target for BMS management (27). To the best of our knowledge, only one previous study has evaluated racial/ethnic disparities related to BMS (28). In a recent systematic review, Wu et al. (5) noted that racial and ethnic disparities related to chronic pain have been widely studied; however, there were no such reports on BMS. Interestingly, our analysis revealed that BMS was significantly associated with race, with white individuals being 7.3 times more likely to have BMS than multiracial participants. In a previous study, participants were predominantly white, which was identified as a limitation, as the findings may not be generalizable to more diverse populations (28). This association may be explained by differences in socioeconomic and medical conditions among older adults (5). Thus, this topic deserves further research, particularly given the impact of BMS on quality of life in this population. Xerostomia was reported by 8 participants (44.4%) with BMS, and 7 of these (87.5%) were female, which is consistent with previous studies (15 , 29). Ten participants (55.5%) showed no signs or reported symptoms of dry mouth. Xerostomia accounts for 44% of the adverse reactions and side effects caused by antihypertensive drugs. Moreover, although a significant association between BMS and xerostomia was observed, causality cannot be inferred. A recent study investigated the relationship between xerostomia and pain sensitivity in patients with BMS and found differential effects across pain dimensions. The authors reported no significant change in the experimentally obtained pain threshold, while self-assessed suprathreshold pain intensity increased (30). No participant with BMS reported smoking during the interview; however, three of them (16.6%) stated that they were former smokers, as shown in Table 1. In total, 41 participants used complete or partial dental prostheses. All participants with BMS had dental prostheses and/or required oral rehabilitation with prosthetic devices. This study has some limitations. First, as a cross-sectional study, it does not establish a cause-effect relationship between the factors analyzed. Further longitudinal studies are needed to investigate causality. Second, the sample was recruited from elderly healthcare support units in the municipality of Manaus, in northern Brazil, and therefore cannot be assumed to represent the broader population of elderly patients. Third, the use of a numeric pain scale could introduce variability into data interpretation. It is important to note that methodological differences in the literature and the lack of consensus on the diagnosis of BMS cause prevalence rates to vary, making comparisons between this study's results and previous findings difficult. The construction of the diagnostic criteria and the factors related to BMS are consistent with the literature, the research lines, and the specific group of elderly people selected for this study. In conclusion, race and xerostomia were associated with the occurrence of BMS. These findings may support clinicians in refining treatment strategies through a personalized approach for older adults. Further research is warranted to better understand BMS and its associated factors in this population.

## Figures and Tables

**Table 1:** Number (n) and percentage (%) of independent factors for burning mouth syndrome, and results of univariate and binary logistic regression analyses.

Independent variable	Burning Mouth Syndrome		Crude OR (95%CI)	P-value**	Adjusted OR (95%CI)	P-value***
	Yes	No				
Sex						
Male	4 (3.6%)	107 (96.4%)	¾	0.439	¾	¾
Female	14 (5.5%)	240 (94.5%)				
Age group*						
Up to 68 years old	9 (4.5%)	192 (95.5%)	¾	0.648	¾	¾
69 years and over	9 (5.5%)	154 (94.5%)				
Race						
Multiracial	6 (2.7%)	216 (97.3%)	7.0#	< 0.001	7.3#	0.031
White	12 (16.2%)	62 (83.8%)	(2.5-19.3)		(2.4-22.1)	
Black	0 (0.0%)	29 (100.0%)				
Indigenous	0 (0.0%)	18 (100.0%)				
Yellow	0 (0.0%)	2 (100.0%)				
Diabetes						
Yes	8 (11.1%)	64 (88.9%)	3.5	0.013	2.0	0.232
No	10 (3.4%)	283 (96.6%)	(1.3-9.3)		(0.6-6.2)	
Hypertension						
Yes	11 (8.3%)	121 (91.7%)	2.9	0.024	2.2	0.174
No	7 (3.0%)	226 (97.0%)	(1.1-7.8)		(0.7-6.5)	
Heart disease						
Yes	5 (98.4%)	47 (1.6%)	2.5	0.096	1.1	0.897
No	13 (92.6%)	300 (7.4%)	(0,8-7.2)		(0.3-4.1)	
Other diseases						
Yes	12 (9.6%)	183 (90.4%)	¾	0.248	¾	¾
No	6 (3.5%)	164 (96.5%)				
Medicines						
Yes	18 (5.7%)	296 (94.3%)	¾	0.062	¾	0.997
No	0 (0.0%)	51 (100.0%)				
Smoking						
Yes	0 (0.0%)	13 (100.0%)	¾	0.247	¾	¾
No	18 (5.1%)	334 (94.9%)				
Former smoking						
Yes	3 (2.9%)	101 (97.1%)	¾	0.224	¾	¾
No	15 (6.0%)	235 (94.0%)				
Complete prostheses						Complete prostheses
Yes	8 (8.6%)	85 (91.4%)	2.5	0.058	1.8	Yes
No	10 (3.7%)	262 (96.3%)	(0.9-6.4)		(0.6-5.6)	No
						
Partial prostheses						Partial prostheses
Yes	6 (5.6%)	101 (94.4%)	¾	0.701	¾	Yes
No	12 (4.7%)	246 (95.3%)				No
Xerostomia						Xerostomia
Yes	8 (12.7%)	55 (87.3%)	4.2	0.005	3.8	Yes
No	10 (3.3%)	292 (96.7%)	(1.6-11.2)		(1.2-11.8)	No

1

## Data Availability

Data are available from the corresponding author upon reasonable request.
